# GSDAT-Net: Enhancing Image Super-Resolution with Grid-Spatial Dual Attention Hybrid Transformer

**DOI:** 10.3390/s26144634

**Published:** 2026-07-22

**Authors:** Yunqiang Liu, Ting Wei, Jinhua Wang

**Affiliations:** 1 School of Microelectronics Industry-Education Integration, Lanzhou University of Technology, Lanzhou 730050, China; 2 School of Automation and Electrical Engineering, Lanzhou University of Technology, Lanzhou 730050, China

**Keywords:** image super-resolution, SwinV2 Transformer layer, grid attention, enhanced spatial attention, multi-scale features

## Abstract

**Highlights:**

**What are the main findings?**

- A Grid-Spatial Dual Attention-driven residual hybrid Transformer (GSDAT-Net) is proposed for image super-resolution, which enhances multi-scale local representation and global contextual modeling.
- The proposed method achieves comparable reconstruction performance on standard benchmark datasets, with a maximum PSNR improvement of 0.15 dB over the selected comparison methods.

**What are the implications of the main findings?**

- Demonstrates that coordinated use of spatial attention, window-based contextual modeling, and grid-based interaction enhances local detail recovery and long-range feature aggregation in image super-resolution.
- The proposed hybrid architecture provides a competitive accuracy–complexity trade-off on standard image super-resolution benchmarks.

**Abstract:**

Image super-resolution (SR) is a fundamental task in computer vision that aims to recover high-fidelity details from low-resolution images. To address the limitations of existing Transformer-based SR methods, this paper introduces GSDAT-Net, a grid-spatial dual-attention-driven residual hybrid Transformer. By integrating Grid Attention Block (GAB), an Enhanced Spatial Attention (ESA) module, and SwinV2 Transformer layers (S2TL), GSDAT-Net extracts multi-scale features and improves local and global feature representation. Extensive experiments demonstrate that GSDAT-Net achieves competitive reconstruction accuracy and visual quality compared with the selected methods, with a maximum PSNR improvement of up to 0.15 dB on benchmark datasets.

## 1. Introduction

As a fundamental task in computer vision, Single Image Super-Resolution (SISR) focuses on recovering high-fidelity details from degraded, low-resolution (LR) observations. Beyond its theoretical value, SISR has been investigated as a preprocessing technique in several domain-specific imaging tasks, including medical imaging [1,2], biometric analysis [3,4], and remote sensing [5,6]. However, the present study focuses exclusively on standard natural-image SR benchmarks and does not directly evaluate these application scenarios. By leveraging sophisticated algorithms to estimate missing high-frequency information, SISR provides an effective computational approach for enhancing image reconstruction quality from low-resolution observations.

Super-resolution (SR) is an inherently ill-posed problem because a low-resolution image may produce a correspondingly large number of high-resolution images, which are not in a one-to-one mapping relationship. Previously, researchers mostly used interpolation-based [7], reconstruction-based [8], and learning-based [9] methods to reconstruct super-resolution images, among which learning-based methods mostly utilize neural networks to reconstruct images with improved reconstruction quality.

Convolutional Neural Network (CNN)-based methods have shown strong performance in single-image super-resolution tasks by learning complex nonlinear mappings through deep convolutional architectures. For example, Kim et al. [10] designed the Very Deep Super-Resolution (VDSR) model with twenty convolutional layers by cascading small filters within a deep architecture. Tai et al. [11] proposed a Deep Recursive Residual Network (DRRN), utilizing recursive structures and residual learning to control the parameter footprint while increasing network depth. Furthermore, Lai et al. [12] developed the Deep Laplacian Pyramid Super-Resolution Network (LapSRN), which leverages recurrent layers to share parameters across pyramid levels, thereby reducing parameter redundancy and enhancing reconstruction quality.

To enhance the model’s focus on key regions, attention mechanisms have been widely integrated into SR tasks. For instance, Zhang et al. [13] developed the Deep Residual Channel Attention Network (RCAN), employing long skip connections and channel attention to improve reconstruction accuracy. Similarly, Xu et al. [14] utilized Enhanced Densely Connected Attention Residual Groups to construct the EASR network. Yu et al. [15] introduced the reference-based R-DHAN, which employs a texture transfer backbone (SRNTT) to recover rich details at ×4 magnification. Furthermore, the Progressive Interactive Learning Network (PILN) proposed by Qin et al. [16] refines features across global, intermediate, and local levels, while Park et al. [17] introduced the dynamic DRSAN, which adjusts its structure based on input statistics to strike a balance between complexity and performance.

Following their success in high-level vision and Natural Language Processing (NLP), Transformers have recently been adapted for SR tasks. Chen et al. [18] introduced the Pre-trained Image Processing Transformer (IPT) specifically to handle various low-level visual tasks. Recent advancements have further optimized these architectures for complex image restoration scenarios. For instance, the Efficient Attention Pyramid Transformer (EAPT) [19] has been proposed to enhance feature representations in general image processing, while dual-branch networks based on Swin Transformers, such as SCNet [20], have demonstrated strong capabilities in structural detail recovery. Additionally, Choi et al. [21] incorporated N-Gram contexts into Transformers, employing a sliding-window self-attention mechanism to expand the receptive field for degraded-pixel recovery. Despite these advancements, Transformer-based SR methods face several limitations. Current window-based architectures typically confine self-attention computation to non-overlapping local windows. This restriction limits the scope of feature aggregation and frequently leads to blocking artifacts or structural inconsistencies, thereby hindering the overall quality of the reconstructed image.

To solve the above problems, this paper proposes GSDAT-Net, a Grid-Spatial Dual Attention-driven residual hybrid Transformer for image super-resolution, which effectively fuses multi-scale features to extract rich contextual information. Based on the Swin Transformer architecture, the proposed algorithm incorporates S2TL to strengthen long-range dependency modeling and improve contextual feature aggregation for richer feature representation. Furthermore, a Fusion Attention Block (FAB) combining the S2TL with ESA is designed, aiming to capture high-frequency details of an image more efficiently by leveraging both local and non-local priors. Based on the attention mechanism and receptive field optimization, grid attention is introduced to jointly model similar features across neighborhoods via the hierarchical extraction of channel dimensions for comprehensive cross-neighborhood information interaction.

The main contributions of our GSDAT-Net are summarized as follows.

(1) We adapt the shifted-window self-attention mechanism of the original STL and design the S2TL module to enhance contextual representation capability and expand the effective attention range, thereby improving the network’s ability to represent complex textures and fine structures.
(2) We design a Fusion Attention Block (FAB) by embedding the ESA before the S2TL, which encourages the network to focus on key regions of interest, thereby extracting more informative feature representations and improving high-frequency detail reconstruction.
(3) Finally, grid attention is introduced for cross-neighborhood information interaction, which compensates for the limitation of S2TL that restricts the self-attention computation to non-overlapping windows. This integration extends the feature aggregation range and enhances multi-scale feature extraction, improving reconstruction quality.

## 2. Related Works

### 2.1. CNN-Based SISR

Driven by the rapid advancement of deep learning [22], Dong et al. [23] pioneered a CNN-based SISR algorithm, achieving significant advancements in restoring image texture details. Despite advancements in accuracy, this method suffers from slow convergence and high training costs. To break the computational bottleneck and extract more accurate deep-level information, Tian et al. [24] employed an enhanced group convolutional neural network to achieve image super-resolution. Additionally, Dun et al. [25] proposed a multi-channel fusion block and a kernel engagement block to restore rich textural information and enhance the network’s representational capacity for multiple kernels. Ran et al. [26] introduced a universal CNN fusion framework with high-resolution guidance, achieving improved spatial quality while preserving spectral information. These methods have made significant progress in enhancing image reconstruction quality; however, they often fall short in multi-scale feature fusion and image detail restoration, limiting their effectiveness in recovering complex image details.

### 2.2. Transformer-Based SISR

In recent years, Transformer-based SISR has become an emerging research direction in super-resolution. Specifically, Liang et al. [27] proposed SwinIR based on the Swin Transformer, which employs a shifted window mechanism for long-range dependency modeling, reducing the number of parameters while achieving impressive visual results. Chen et al. [28] utilized alternating spatial and channel attention in consecutive Transformer blocks, aggregating inter-block features to enhance the extraction of high-frequency details. Rossi et al. [29] upgraded the Swin2SR model by introducing an enhanced sparse-gated hybrid expert layer to replace the Transformer’s internal feed-forward network, thereby enhancing overall performance. Building upon such efficient gating mechanisms, multi-scale gated networks [30] have also been recently developed to achieve an optimal balance between image restoration quality and computational cost. Fang et al. [31] proposed a lightweight hybrid network combining CNN and Transformer architectures, leveraging both local and non-local priors to extract beneficial features for SR. This hybrid design philosophy has similarly driven the development of hybrid attention separable networks (HASN) [32] to further optimize detail recovery. Finally, Chu et al. [33] introduced a novel hybrid multi-axis aggregation network to better explore latent feature information.

Pixel-level attention provides another effective strategy for spatially non-uniform image super-resolution. The Pixel Attention Network generates three-dimensional attention maps to adaptively recalibrate individual spatial-channel features, enabling different image regions to receive content-dependent responses [34]. Meanwhile, structured doubly stochastic similarity learning constrains an affinity matrix through non-negativity and balanced row- and column-wise normalization, thereby reducing excessively concentrated or one-sided correspondences [35]. Although the latter was originally developed for graph-based learning rather than SR, its balanced correspondence principle may inspire future pixel- or patch-level attention designs for non-uniform reconstruction.

In addition to deterministic CNN- and Transformer-based methods, normalizing flows provide a probabilistic alternative for image super-resolution. SRFlow explicitly learns the conditional distribution of plausible high-resolution images given a low-resolution input, thereby addressing the one-to-many nature of the SR problem [36]. HCFlow further models the low-frequency image and the remaining high-frequency information in a hierarchical conditional framework, enabling both image super-resolution and image rescaling [37]. Compared with these probabilistic methods, GSDAT-Net focuses on deterministic reconstruction accuracy by enhancing local, global, and cross-neighborhood feature interaction.

## 3. Proposed Method

In this section, we first delineate the overall network architecture and then detail its internal components.

### 3.1. Network Architecture

**Figure 1:**
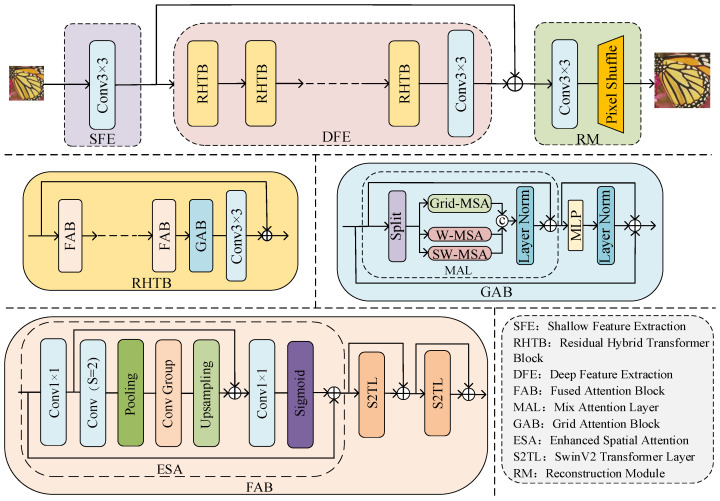
Overall network architecture diagram.

The network in this paper consists of three main parts: shallow feature extraction, deep feature extraction based on the Residual Hybrid Transformer Block (RHTB), and an image reconstruction module. As shown in Figure 1, the input image is initially converted into shallow features for subsequent deep feature extraction. Then, the extracted shallow features are fed into stacked RHTBs to extract and fuse deeper features. Finally, a pixel-shuffle layer is used to resize the image to a predetermined resolution and generate a reconstructed image. The designed RHTB contains two core sub-blocks: the FAB and the GAB. Specifically, the FAB comprises an ESA module and an S2TL. The ESA module is deployed at the front-end of the FAB to enhance the capability of capturing high-frequency features, while the S2TL is used for information exchange between non-overlapping windows to extract rich contextual information. The GAB is used to interact with information across neighborhoods to jointly model similar features to increase the effective receptive field and enhance the multi-scale feature extraction capability of the network. The detailed workflow of our method is summarized in Algorithm 1.
**Algorithm 1:** Training procedure of the proposed GSDAT-Net for image super-resolution**Input**: Low-resolution training image $I_{LR}$, corresponding high-resolution image $I_{HR}$, scale factor s, network parameters θ.**Output**: Super-resolved image $I_{SR}$ and optimized model parameters θ.1:    Extract shallow features $F_{s}$ from $I_{LR}$ according to Equation (1). 2:    **for** d = 1 to D **do** 3:     Feed the input feature into the d-th RHTB. 4:     Apply ESA to enhance spatially important local features. 5:     Apply S2TL to model local and shifted-window contextual dependencies. 6:     Apply GAB to establish grid-based cross-neighborhood feature interaction according to Equations (4)–(11). 7:     Fuse the features through convolution and residual connection. 8:    **end for** 9:    Obtain deep features $F_{d}$ from the stacked RHTBs according to Equation (2). 10: Aggregate $F_{s}$ and $F_{d}$ using global residual learning. 11: Generate $I_{SR}$ through the pixel-shuffle reconstruction module according to Equation (3). 12: Compute the reconstruction loss between $I_{SR}$ and $I_{HR}$. 13: Optimize θ using back-propagation. 14: Return $I_{SR}$.

To provide a rigorous overview, the network workflow is mathematically formulated below. First, during the shallow feature extraction stage, the input low-resolution image is mapped into a high-dimensional feature space via a 3 × 3 convolutional layer, derived as follows:

(1) $$F_{0}=f_{SF}(I_{LR})$$

where $f_{SF}$ represents the convolution operation, the extracted shallow features $F_{0}$ will be further used for deeper feature extraction. Meanwhile, the features $F_{0}$ are directly transferred to the reconstruction module through a residual connection to reduce the loss of low-level features and generate more accurate edge features. Subsequently, the shallow features $F_{0}$ are fed as input to the successive stacked RHTBs to extract the deep feature information. Specifically, a 3 × 3 convolution and six RHTBs form the deep feature group, and each RHTB consists of three FABs, a GAB, and a convolution layer with residual connections, and the operation of the RHTB for extracting the deep features is shown in Equation (2):

(2) $$F_{D}=f_{RHTB}^{d}(f_{RHTB}^{d-1}\cdots ((f_{RHTB}^{1}(F_{0}))))+f_{CONV}(F_{d})\;(d=1,2,\ldots ,6)$$

where $f_{RHTB}^{d}$ denotes the *d*th RHTB operation for extracting the depth features of the image. Finally, an element-wise summation of the features $F_{0}$ and $F_{D}$ is input to the reconstruction module through residual linking, and the feature map is resized to the pixel size of the high-resolution image using a pixel-shuffle layer:

(3) $$I_{SR}=f_{REC}(F_{D}+F_{0})$$

where $I_{SR}$ is the final generated reconstructed image and $f_{REC}$ is the upsampling layer.

### 3.2. Grid Attention Block

To improve image reconstruction, a GAB is introduced to enhance the network’s capacity for multi-scale feature extraction via hierarchical partitioning of channel dimensions, thereby increasing the effective receptive field. The GAB consists of a Mixed Attention Layer (MAL) and a Multilayer Perceptron (MLP). The three attention branches play complementary roles in feature aggregation. W−MSA models fine-grained dependencies among tokens within each fixed local window, whereas SW−MSA enables information exchange between neighboring windows through shifted partitioning. In contrast, Grid−MSA rearranges tokens according to regularly spaced grid positions and computes attention among spatially separated regions, thereby establishing cross-neighborhood interactions beyond adjacent windows. Unlike standard global self-attention, which directly models pairwise relations among all spatial tokens and incurs quadratic complexity with respect to image size, the proposed mixed-attention design restricts computation to structured window and grid groups. Consequently, it enlarges the effective feature aggregation range while maintaining a more manageable computational cost. For MAL, the input features $F_{in}$ are first split along the channel dimension into two components: $F_{G}\in R^{H\times W\times \frac{C}{2}}$ and $F_{W}\in R^{H\times W\times \frac{C}{2}}$. Subsequently, $F_{W}$ is further partitioned along the channels to serve as inputs for W−MSA and SW−MSA, respectively, while $F_{G}$ is routed into Grid−MSA. The MAL is calculated as follows:

(4) $$X_{W_{1}}=W-MSA(F_{W_{1}})$$

(5) $$X_{W_{2}}=SW-MSA(F_{W_{2}})$$

(6) $$X_{G}=Grid-MSA(F_{G})$$

(7) $$X_{MAL}=LN(Cat(X_{w_{1}},X_{w_{2}},X_{G}))+F_{in}$$

where $X_{w_{1}},{\;X}_{w_{2}},\;and\;X_{G}$ represent the output features of W−MSA, SW−MSA, and Grid−MSA, respectively. To enhance the stability of network training, this paper adopts the post-normalization paradigm within the GAB. For a given input $F_{in}$, the GAB workflow is computed as follows:

(8) $$F_{M}=LN(MAL(F_{in}))+F_{in}$$

(9) $$F_{out}=LN(MLP(F_{M}))+F_{M}$$

**Figure 2:**
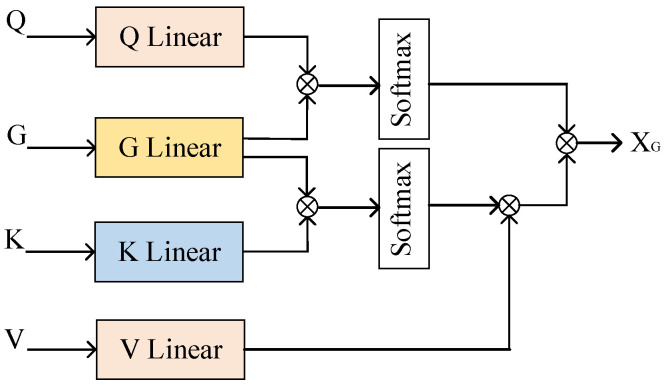
Flowchart of grid attention calculation.

As shown in Figure 2, when deploying Grid−MSA, the query (Q), key (K), and value (V) matrices are derived from the input features $F_{G}$ that have been rearranged by the grid. Specifically, the grid feature $G\in R^{H\times W\times \frac{C}{2}}$ is obtained from the input features $F_{in}$ by linear transformation followed by grid shuffling. For Grid-MSA, the self-attention mechanism is mathematically formulated as follows:

(10) $$\hat{X}=SoftMax(\frac{GK^{T}}{d}+B)V$$

(11) $$Attention(Q,G,\hat{X})=SoftMax(\frac{QG^{T}}{d}+B)\hat{X}$$

where $\hat{X}$ is the intermediate feature obtained by computing the self-attention of G, K, and V.

### 3.3. Enhanced Spatial Attention Module

Simulating the human visual system, attention mechanisms can direct a network’s computational resources toward key informative regions. To further improve the model’s feature extraction capability, this paper adopts the ESA [38], which concentrates on critical regions of interest more effectively than conventional spatial attention mechanisms [39], thereby aggregating highlighted features to yield more discriminative representations. Given an input $F_{in}^{esa}$, ESA first extracts compact features $F_{1}^{esa}$, as follows:

(12) $$F_{1}^{esa}=W_{1}^{esa}*F_{in}^{esa}$$

where ∗ denotes the convolution operation, and $W_{1}^{esa}$ represents the weight of the 1 × 1 convolutional layer deployed to reduce the channel dimensionality.

Then, the ESA further extracts features $F_{2}^{esa}$, as follows:

(13) $$F_{2}^{esa}=H_{up}(H_{g}(H_{pool}(W_{2}^{esa}*F_{1}^{esa})))$$

where $W_{2}^{esa}$ represents the weight of the 3 × 3 convolution with a stride of 2, $H_{pool}$ signifies the maximum pooling operation, $H_{g}$ denotes a compound function consisting of three 3 × 3 convolutional layers, and $H_{up}$ is the upsampling function implemented via bilinear interpolation. Through this design, the spatial dimensions are reduced by the convolutional and maximum pooling layers and subsequently restored by the upsampling layer to preserve structural integrity. Finally, the ultimate output,${\;F}_{out}^{esa}$, of the ESA module is evaluated as follows:

(14) $$F_{out}^{esa}=H_{sigmoid}\left(W_{3}^{esa}*(F_{1}^{esa}+F_{2}^{esa})\right)\times F_{in}^{esa}$$

where $W_{3}^{esa}$ is the weight of the 1 × 1 convolutional layer used to recover the embedding dimension, $H_{sigmoid}$ represents the sigmoid function, and the symbol × denotes the element-wise multiplication operation.

### 3.4. Swin-Based Transformer Layer

#### 3.4.1. Swin Transformer Layer

**Figure 3:**
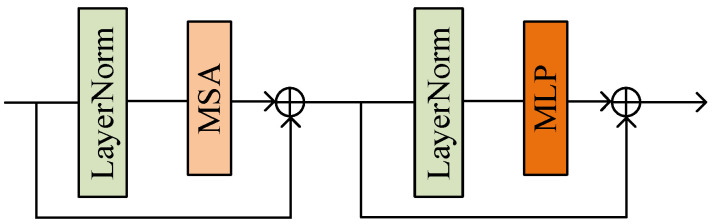
STL internal structure diagram.

The internal structure of STL is shown in Figure 3. The Swin Transformer is a widely adopted vision Transformer architecture based on the self-attention mechanism, which processes images in non-overlapping patches to reduce computational complexity while maintaining high reconstruction accuracy. The Swin Transformer adopts a cross-stage information exchange mechanism, allowing the model to capture multi-scale features to construct a global feature representation.

Given an input tensor of size H×W×C, the network first reshapes the feature maps into a dimension of $\frac{HW}{M^{2}}\times M^{2}\times C$ by partitioning the input into non-overlapping M×M localized windows, where $\frac{HW}{M^{2}}$ denotes the total number of windows. Subsequently, standard self-attention (i.e., local attention) is computed independently within each localized window. For a localized window feature $X\in R^{M^{2}\times C}$, the query, key, and value matrices Q, K, and V are computed as follows:

(15) $$Q=XP_{Q},\;K=XP_{K},\;V=XP_{V}$$

where $P_{Q}$,$\;P_{K}$, and $P_{V}$ are the projection matrices shared across different windows, and $Q,K,V\in R^{M^{2}\times d}$. The attention matrix Attention(Q,K,V) within a local window is computed via the self-attention mechanism using the following expression:

(16) $$Attention(Q,K,V)=SoftMax{(}^{QK^{T}}/\sqrt{d}+B)V$$

where B represents the learnable relative positional encoding matrix, and d denotes the dimensionality of the multi-head attention mechanism.

Furthermore, the Multilayer Perceptron (MLP) incorporates two fully connected layers equipped with GELU nonlinearities for subsequent feature transformation. The LayerNorm (LN) layer is prefixed before both the MSA and MLP, and both modules employ residual connections, which are mathematically structured as follows:

(17) X=MSA(LN(X))+X

(18) X=MLP(LN(X))+X

The Swin Transformer module utilizes regular and shifted window partitioning configurations of window-based self-attention to achieve cross-window connectivity, which facilitates information exchange among non-overlapping windows to expand the network’s receptive field.

#### 3.4.2. SwinV2 Transformer Layer

**Figure 4:**
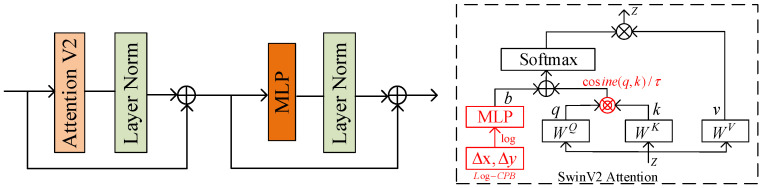
S2TL internal structure diagram.

To improve contextual modeling capability and enlarge the effective attention range, this paper presents S2TL, whose detailed architecture is illustrated in Figure 4. Compared with the conventional STL, the S2TL restructures the shifted-window self-attention mechanism and enhances information exchange among local windows, thereby enabling richer contextual feature interaction. Specifically, adopting a post-normalization paradigm instead of the conventional pre-normalization effectively mitigates feature amplitude explosion in deep layers, thereby significantly improving numerical stability during training. Furthermore, employing scaled cosine attention instead of the standard dot-product self-attention reduces the risk that attention heads are dominated by limited pixel correlations, encouraging more balanced feature learning. For the configuration of the S2TL, this paper retains the window size, number of channels, and number of attention heads settings of the original STL, which are 8, 180, and 6, respectively. The updated self-attention is computed as in Equation (20), and the specific operation is shown below:

(19) Attention(Q,K,V)=SoftMax(cos(Q,K)/τ+S)V

where $S\in R^{M^{2}\times M^{2}}$ denotes the continuous relative position bias matrix, and τ represents a learnable scalar.

### 3.5. Fusion Attention Module

The complementary advantages of global and local modeling can effectively improve the quality of the reconstructed image. Therefore, this paper embeds an enhanced spatial attention module before the SwinV2 Transformer layer to strengthen the network’s ability to capture high-frequency features while promoting comprehensive global information fusion. Mathematically, the forward propagation within this stage is formulated as follows:

(20) $$F_{F}=H_{F}(F_{F_{m}})+F_{F_{m}}$$

where $F_{F_{m}}$ denotes the input features and $F_{F}$ denotes the output features from the ESA module.

In this paper, we follow the STL in the SwinIR architecture to design the S2TL, including window-based self-attention (W-MSA), shifted window-based multi-head self-attention (SW-MSA), and Layer Normalization (LN). The operational procedure of the updated S2TL is mathematically expressed via Equations (21) and (22):

(21) $$F_{N}=(S)W-LN(AttentionV2(F_{W_{in}}))+F_{W_{in}}$$

(22) $$F_{out}=LN(MLP(F_{N}))+F_{N}$$

where $F_{W_{in}}$, $F_{N}$, and $F_{out}$ denote input features, intermediate representations, and output features, respectively.

## 4. Experiments

### 4.1. Datasets

The experiments use the publicly available DIV2K [40] dataset as the training dataset, which contains 800 high-resolution (HR) images with a resolution of 2K, and the LR images are generated from HR images using bicubic downsampling following the standard degradation protocol adopted by SwinIR and other Transformer-based SR methods. To extend the dataset, data augmentation is performed on both HR and LR images across different scale factors using horizontal flipping and random rotations. Finally, these images were cropped into image blocks of size 64×64 respectively for model training.

The widely used benchmark datasets—Set5 [41], Set14 [42], BSD100 [43], Urban100 [44], and Manga109—are adopted as the test sets and contain 5, 14, 100, 100, and 109 images, respectively, covering images of various scenarios ranging from natural landscapes to complex urban structures. To evaluate the performance of the proposed algorithm, Peak Signal-to-Noise Ratio (PSNR) [45] and Structural Similarity (SSIM) [46] are used as the evaluation metrics for this experiment, and the quantitative evaluation is performed exclusively on the Y channel of the YCbCr color space.

### 4.2. Implementation Details

The proposed network is evaluated within a unified hardware and software environment configured as follows: an Ubuntu 22.04.2 operating system, an Intel (R) Xeon (R) Gold 5220R CPU @2.20GHZ, 256 GB of RAM, and an NVIDIA GeForce 3090 GPU. All models are implemented using the PyTorch (version 2.0.1+cu117) deep learning framework and programmed in Python 3.8. The $L_{1}$ loss is adopted as the objective function to optimize the network parameters. We utilize the Adam optimizer for model optimization, with its hyperparameters set to $\beta_{1}$ = 0.9, $\beta_{2}$ = 0.99. During the training phase, the batch size is 32. The initial learning rate is set to 0.0002, which is decayed by a factor of 0.5 at the 250,000th, 400,000th, 450,000th, and 475,000th iterations, respectively. To ensure the reproducibility of the experiments, a fixed random seed of 1234 is used for all experiments, including network initialization, data shuffling, and augmentation operations. The reported results are obtained under this fixed seed setting.

### 4.3. Comparison with State-of-the-Art Methods

#### 4.3.1. Quantitative Comparison

After the model training is completed, the LR images in the test dataset are reconstructed with super-resolution, and the comparison test is performed on the four benchmark test sets with magnification factors of ×2, ×3, and ×4. The comparison of the quantitative results is given in Table 1, Table 2 and Table 3, where the bolded ones are the optimal results, and the underlined ones are the sub-optimal ones. As can be seen from Table 1, Table 2 and Table 3, compared to SRCNN [23], CARN [47], IMDN [48], LatticeNet [49], A2N [50], SwinIR [27], ESRT [51], HNCT [31], PILN [16], DRSAN [17], Omni-SR [52], IFIN [53], and CAMixerSR [54] several current State-of-the-Art super-resolution algorithms, the PSNR and SSIM of this paper’s algorithm are achieves comparable or slightly better performance than selected methods. Specifically, compared with the CAMixerSR method in the recently published CVPR2024 paper, the PSNR is improved by 0.15, 0.14, and 0.02 dB on the Set5 test set with amplification factors of ×2, ×3, and ×4, respectively; and the PSNR is improved by 0.13, 0.05, and 0.06 dB on the BSD100 test set with amplification factors of ×2, ×3, and ×4, respectively. Meanwhile, horizontally, the optimal PSNR and SSIM are obtained on all four test sets when the amplification factor is ×2. Specifically, compared with CAMixerSR, the PSNR is improved by 0.15 dB, 0.11 dB, 0.13 dB, and 0.17 dB, while the SSIM increases by 0.0013, 0.0012, 0.0013, and 0.0026 on Set5, Set14, BSD100, and Urban100, respectively. This improved performance is primarily attributed to the expanded receptive field offered by our grid attention mechanism, which works in tandem with the S2TL feature extraction unit to strengthen the network’s understanding of long-range contextual relationships and effectively facilitate the reconstruction of high-frequency textures.

Nevertheless, the proposed algorithm yields sub-optimal results on the Urban100 dataset at ×3 and ×4 upscaling factors. This performance characteristic is primarily because Urban100 is characterized by highly dense, repetitive geometric patterns and distinct structural styles, which inherently challenge the inductive biases of different network paradigms. Consequently, individual reconstruction algorithms exhibit varying capabilities when processing specific image characteristics, leading to minor performance fluctuations under challenging high-magnification scales.

#### 4.3.2. Visual Comparison

**Figure 5:**
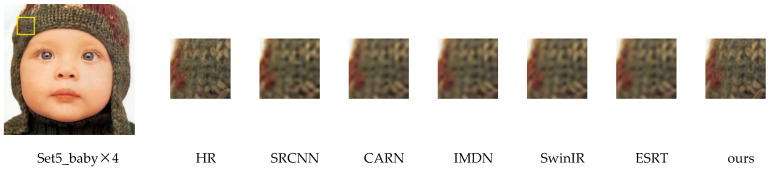
Comparison of the reconstruction effects of different algorithms on the baby in the Set5 test set under a ×4 magnification factor.

**Figure 6:**
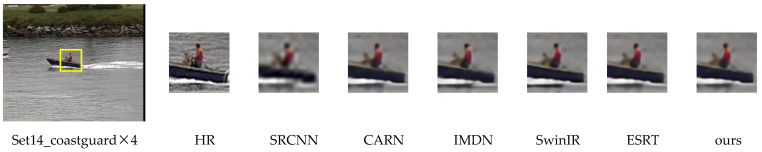
Comparison of the reconstruction effects of the coastguard in the Set14 test set by different algorithms under a ×4 magnification factor.

**Figure 7:**
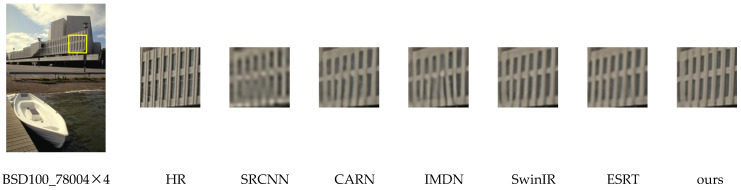
Comparison of the reconstruction effects of the 78,004 in the BSD100 test set by different algorithms under a ×4 magnification factor.

**Figure 8:**
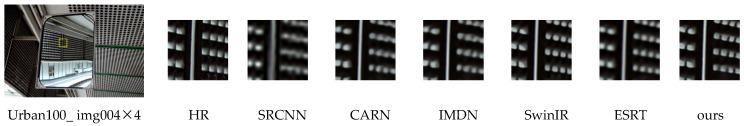
Comparison of the reconstruction effects of img004 in the Urban100 test set by different algorithms under a ×4 magnification factor.

To further validate the qualitative advantages of the network, some representative images tested in the benchmark dataset are analyzed by visualizing the results. In Figure 5, the baby images in the Set5 test set are reconstructed using different algorithms, where the cropped regions highlighted by yellow rectangles are magnified for detailed visual comparison. It can be seen that the images reconstructed by SRCNN, CARN, IMDN, SwinIR, and ESRT methods suffer from noticeable blurring artifacts, while the images reconstructed by the algorithms in this paper have a clearer texture of the wool details, and the contours are also clearer and appear more realistic, which is the closest to the real HR images. In Figure 6, the Set14 test set of the coastguard image, compared to smooth image areas, recovering high-frequency features in non-smooth edge structures with rapid spatial changes, presents a significantly higher technical challenge. Visual close-ups of the cropped patches reveal that existing methods exhibit limited capabilities in preserving structural integrity, whereas this paper’s method reduces the distortion, blurring artifacts, and other defects, showing better reconstruction results. In Figure 7, concerning the enlarged details of the texture of the building in BSD100, the image reconstructed by several other mainstream algorithms suffers from severe geometric distortion and structural deformation, while the image reconstructed by the model in this paper effectively suppresses these undesirable artifacts, accurately recovering crisp structural grids and delivering enhanced visual fidelity. For the “img004” image from the Urban100 dataset shown in Figure 8, visual close-ups demonstrate that GSDAT-Net reconstructs sharper geometric angular structures and crisp rectilinear grids, avoiding the shape distortion prevalent in competing methods. The improved visual quality of our network stems from the interactive feature fusion within the ESA and S2TL modules. This cooperative design enhances the network’s ability for multi-scale feature extraction, enabling the explicit capture of both local texture similarities and global structural correlations across the entire image. More importantly, the cross-neighborhood information interaction provided by our grid attention mechanism successfully mitigates the inherent limitation of S2TL, which restricts self-attention computations to non-overlapping local windows, thereby extending the feature aggregation range to recover authentic high-frequency details.

### 4.4. Ablation Study

To validate the effectiveness of the proposed network architecture for image reconstruction, ablation studies on ESA and GAB modules were conducted on the Urban100 dataset under a ×4 upscaling factor. The comparative network performance results are presented in Table 4. Experimental results demonstrate that the optimal performance is achieved when both modules are jointly employed. The performance gains obtained by using either ESA or GAB individually are suboptimal relative to their combined integration. Specifically, the standalone ESA module exhibits a marginally superior performance over its GAB counterpart. Nevertheless, the GAB module effectively facilitates cross-neighborhood information interaction, significantly enhancing PSNR/SSIM metrics and better restoring image textural details. These ablation studies demonstrate the contributions of the proposed modules to the algorithm’s efficacy.

As shown in Table 5, replacing the original STL with the proposed S2TL improves reconstruction accuracy while maintaining a comparable parameter scale. In addition, placing ESA before S2TL achieves better performance than placing it after S2TL. This indicates that emphasizing informative spatial and high-frequency features before window-based contextual modeling is more beneficial for SR reconstruction.

As shown in Table 6, as the number of RHTBs increases, reconstruction performance gradually improves due to enhanced feature extraction capability. However, additional RHTBs introduce more parameters and computational cost. Considering the trade-off between accuracy and complexity, six RHTBs are adopted in GSDAT-Net.

As shown in Table 7, smaller windows restrict contextual interaction, whereas excessively large windows increase computational cost. A window size of 8 achieves a better balance between reconstruction performance and computational complexity.

### 4.5. Model Complexity and Efficiency Analysis

In addition to the evaluation of the method performance, this paper compares the parameter efficiency and model complexity of the proposed algorithm with seven representative algorithms, namely CARN [47], IMDN [48], LatticeNet [49], SwinIR [27], ESRT [51], Omni-SR [52] and CAMixerSR [54] under the ×4 upscaling factor, and the parameters, FLOPs, runtime, GPU memory consumption, and architecture used for each method are given in Table 8.

For a fair efficiency comparison, all models were evaluated using the same benchmarking protocol on a single NVIDIA GeForce RTX 3090 GPU. The input was a single low-resolution RGB image of size 256 × 256 pixels, and all models were tested at the ×4 upscaling factor with a batch size of 1 and FP32 precision. Before runtime measurement, each model was executed for 50 warm-up iterations to stabilize CUDA initialization and GPU execution. The inference time was then averaged over 200 forward passes. FLOPs were calculated using the same 128 × 128 low-resolution input, while GPU memory consumption was recorded as the peak allocated memory during inference. Gradient computation and test-time self-ensemble were disabled for all models.

**Figure 9:**
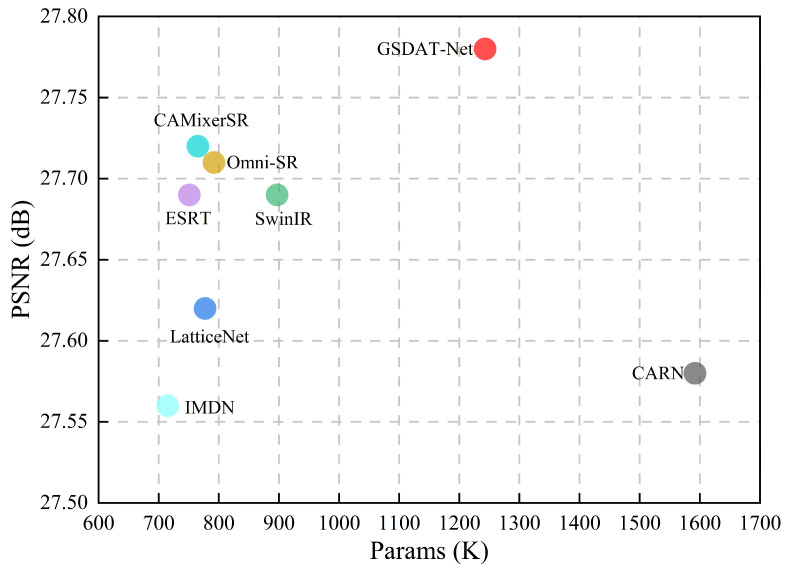
Comparison of parameter efficiency and reconstruction accuracy on BSD100 for ×4 SR.

As shown in Table 8, GSDAT-Net has fewer parameters than CARN but requires more parameters and computational cost than several lightweight methods. However, as illustrated in Figure 9, GSDAT-Net achieves the highest PSNR among the compared methods, demonstrating a favorable trade-off between reconstruction accuracy and model complexity.

Figure 9 further illustrates the parameter-accuracy trade-off on BSD100 under the ×4 setting. The horizontal axis denotes the number of model parameters, and the vertical axis denotes PSNR. Compared with lightweight CNN-based methods, GSDAT-Net requires more parameters, but it achieves a higher reconstruction accuracy. Compared with larger or comparable models such as CARN and SwinIR, GSDAT-Net obtains a better PSNR with a moderate parameter scale. These results indicate that the proposed local-global hybrid attention design provides an effective balance between model complexity and reconstruction quality.

## 5. Discussion

This work develops GSDAT-Net, an improved single-image super-resolution reconstruction network, and comprehensively validates its reconstruction capability on standard benchmark datasets. Quantitative results across mainstream testing scenarios demonstrate that the proposed method outperforms recent State-of-the-Art SR models, including SwinIR, Omni-SR and CAMixerSR, in terms of PSNR and SSIM under standard upscaling factors. Evaluated on the widely adopted Set5, Set14, BSD100, and Urban100 datasets, GSDAT-Net achieves competitive numerical performance, especially at the ×2 upsampling scale, where favorable metric values are obtained on most test subsets. Consistent improvements under standard upscaling factors further confirm the stable and reliable reconstruction performance of the presented method in objective quantitative evaluation.

The performance improvements benefit from the rational structural design and efficient feature modeling of embedded ESA and GAB modules. The introduced S2TL feature extraction unit enhances long-range contextual dependency modeling ability, while the cross-neighborhood interactive strategy of grid attention remedies the inherent feature-aggregation deficiency of conventional window-based self-attention. Jointly, these components enable the network to precisely capture high-frequency edge features and subtle texture details latent in complex images. Visual intuitive ablation and contrast tests further verify the effectiveness of the core modules. Compared with existing CNN-Transformer hybrid algorithms, GSDAT-Net significantly alleviates severe reconstruction artifacts, including edge blurring, texture distortion and structural dislocation, producing high-fidelity visual results that are highly consistent with real HR reference images.

Nevertheless, the proposed method still faces certain practical constraints. Under ×3 and ×4 challenging high-magnification conditions, the model yields slightly limited performance on certain intricate urban scenes within the Urban100 dataset. This is mainly due to distinct style discrepancies across datasets and uneven feature reconstruction preferences of different network paradigms. Moreover, compared to ultra-lightweight pure CNN methods, the cascaded dual-module design inevitably increases the computational and parameter overhead.

In addition, GSDAT-Net currently learns a deterministic mapping from LR to HR images and therefore does not explicitly model the diversity of plausible HR solutions. Normalizing-flow-based methods, such as SRFlow and HCFlow, offer a probabilistic alternative by learning the conditional distribution of HR images. Furthermore, inspired by pixel attention and structured doubly stochastic similarity learning, introducing balanced row- and column-wise constraints into grid attention may improve pixel- or patch-level interaction for spatially non-uniform image reconstruction.

In future research, we will investigate probabilistic image restoration approaches, such as normalizing-flow-based distribution modeling and doubly stochastic attention constraints, while also exploring efficient model optimization strategies, including network pruning, adaptive attention mechanisms, and low-computation feature fusion approaches to reduce computational complexity and inference latency while maintaining reconstruction performance.

## 6. Conclusions

In this paper, we propose GSDAT-Net, a grid-spatial dual attention-driven residual hybrid Transformer for image super-resolution. Specifically, the algorithm designs several RHTBs, and in each block, by integrating enhanced spatial attention, a SwinV2 Transformer layer, and grid attention, it can efficiently extract multi-scale hierarchical features, fuse feature information from different receptive fields, and obtain broader image context information. The experimental results show that the improved algorithm in this paper has a greater improvement in PSNR and SSIM compared with the current State-of-the-Art methods across upscaling factors of ×2, ×3, and ×4, and the reconstructed image texture is clear and visually effective. However, the model in this paper requires a large amount of computational resources during the training process. Therefore, future work will conduct lightweight research on image super-resolution reconstruction networks to reduce computational requirements.

## Figures and Tables

**Table 1:** Performance evaluation metrics of different image reconstruction algorithms on five test sets (magnification of 2).

Method	Publication Year	Set5		Set14		BSD100		Urban100		Manga109	
		PSNR/dB	SSIM	PSNR/dB	SSIM	PSNR/dB	SSIM	PSNR/dB	SSIM	PSNR/dB	SSIM
SRCNN [23]	2016	36.66	0.9542	32.45	0.9067	31.36	0.8879	29.50	0.8946	35.60	0.9663
CARN [47]	2018	37.76	0.9590	33.52	0.9166	32.09	0.8978	31.92	0.9256	38.36	0.9765
IMDN [48]	2019	38.00	0.9605	33.63	0.9177	32.19	0.8996	32.17	0.9283	38.88	0.9774
LatticeNet [49]	2020	38.15	0.9610	33.78	0.9193	32.25	0.9005	32.43	0.9302	—	—
A2N [50]	2021	38.06	0.9608	33.75	0.9194	32.22	0.9002	32.43	0.9311	38.87	0.9769
SwinIR [27]	2021	38.14	0.9611	33.86	0.9206	32.31	0.9012	32.76	0.9340	39.12	0.9783
ESRT [51]	2022	38.03	0.9600	33.75	0.9184	32.25	0.9001	32.58	0.9318	—	—
HNCT [31]	2022	38.08	0.9608	33.65	0.9182	32.22	0.9001	32.22	0.9294	38.87	0.9774
PILN [16]	2023	38.08	0.9607	33.72	0.9181	32.23	0.9003	32.38	0.9306	—	—
DRSAN [17]	2023	38.13	0.9610	33.72	0.9189	32.24	0.9009	32.41	0.9312	—	—
Omni-SR [52]	2023	38.22	0.9613	33.98	0.9210	32.36	0.9020	33.05	0.9363	39.28	0.9784
IFIN [53]	2024	38.00	0.9606	33.66	0.9181	32.18	0.8996	32.14	0.9284	38.95	0.9777
CAMixerSR [54]	2024	38.23	0.9613	34.00	0.9214	32.34	0.9016	32.95	0.9348	—	—
GSDAT-Net	**/**	**38.38**	**0.9626**	**34.11**	**0.9222**	**32.47**	**0.9029**	**33.12**	**0.9374**	**39.30**	**0.9789**

Notes: The bold values indicate the best performance, while the underlined values indicate the second-best performance among all compared methods. The results of SRCNN, CARN, IMDN, LatticeNet, A2N, SwinIR, ESRT, HNCT, PILN, DRSAN, Omni-SR, IFIN, and CAMixerSR are obtained from their original papers. These results are reported under their corresponding official experimental settings. The GSDAT-Net results are evaluated using our implementation with the same benchmark datasets and evaluation metrics.

**Table 2:** Performance evaluation metrics of different image reconstruction algorithms on five test sets (magnification of 3).

Method	Publication Year	Set5		Set14		BSD100		Urban100		Manga109	
		PSNR/dB	SSIM	PSNR/dB	SSIM	PSNR/dB	SSIM	PSNR/dB	SSIM	PSNR/dB	SSIM
SRCNN [23]	2016	32.75	0.9090	29.29	0.8209	28.41	0.7863	26.24	0.7989	30.48	0.9117
CARN [47]	2018	34.29	0.9255	30.29	0.8407	29.06	0.8034	28.06	0.8493	33.50	0.9440
IMDN [48]	2019	34.36	0.9270	30.32	0.8417	29.09	0.8046	28.17	0.8519	33.61	0.9445
LatticeNet [49]	2020	34.53	0.9281	30.39	0.8424	29.15	0.8059	28.33	0.8538	—	—
A2N [50]	2021	34.47	0.9279	30.44	0.8437	29.14	0.8059	28.41	0.8570	—	—
SwinIR [27]	2021	34.62	0.9289	30.54	0.8463	29.20	0.8082	28.66	0.8624	33.98	0.9478
ESRT [51]	2022	34.42	0.9268	30.43	0.8433	29.15	0.8063	28.46	0.8574	33.95	0.9455
HNCT [31]	2022	34.47	0.9275	30.44	0.8439	29.15	0.8067	28.28	0.8557	33.81	0.9459
PILN [16]	2023	34.39	0.9269	30.34	0.8415	29.08	0.8048	28.09	0.8500	33.68	0.9446
DRSAN [17]	2023	34.50	0.9278	30.39	0.8437	29.13	0.8065	28.33	0.8566	—	—
Omni-SR [52]	2023	34.70	0.9294	30.57	0.8469	29.28	0.8094	**28.84**	**0.8656**	34.22	0.9487
IFIN [53]	2024	34.45	0.9278	30.47	0.8442	29.13	0.8064	28.32	0.8560	34.14	0.9479
CAMixerSR [54]	2024	34.65	0.9295	30.62	0.8471	29.26	0.8093	28.81	0.8645	—	—
GSDAT-Net	**/**	**34.79**	**0.9296**	**30.64**	**0.8478**	**29.31**	**0.8098**	28.81	0.8649	**34.24**	**0.9488**

Notes: The bold values indicate the best performance, while the underlined values indicate the second-best performance among all compared methods. The results of SRCNN, CARN, IMDN, LatticeNet, A2N, SwinIR, ESRT, HNCT, PILN, DRSAN, Omni-SR, IFIN, and CAMixerSR are obtained from their original papers. These results are reported under their corresponding official experimental settings. The GSDAT-Net results are evaluated using our implementation with the same benchmark datasets and evaluation metrics.

**Table 3:** Performance evaluation metrics of different image reconstruction algorithms on five test sets (magnification of 4).

Method	Publication Year	Set5		Set14		BSD100		Urban100		Manga109	
		PSNR/dB	SSIM	PSNR/dB	SSIM	PSNR/dB	SSIM	PSNR/dB	SSIM	PSNR/dB	SSIM
SRCNN [23]	2016	30.48	0.8625	27.50	0.7510	26.89	0.7101	24.52	0.7222	27.58	0.8555
CARN [47]	2018	32.13	0.8937	28.60	0.7806	27.58	0.7349	26.07	0.7837	30.47	0.9084
IMDN [48]	2019	32.21	0.8948	28.58	0.7811	27.56	0.7353	26.04	0.7838	30.45	0.9075
LatticeNet [49]	2020	32.30	0.8962	28.68	0.7830	27.62	0.7367	26.25	0.7873	—	—
A2N [50]	2021	32.30	0.8966	28.71	0.7842	27.61	0.7374	26.27	0.7920	30.67	0.9110
SwinIR [27]	2021	32.44	0.8977	28.75	0.7857	27.69	0.7406	26.48	0.7983	30.92	0.9151
ESRT [51]	2022	32.19	0.8947	28.69	0.7833	27.69	0.7379	26.39	0.7962	30.75	0.9100
HNCT [31]	2022	32.31	0.8957	28.71	0.7834	27.63	0.7381	26.20	0.7896	30.70	0.9112
PILN [16]	2023	32.22	0.8949	28.62	0.7813	27.59	0.7365	26.19	0.7878	30.54	0.9086
DRSAN [17]	2023	32.30	0.8954	28.66	0.7838	27.61	0.7381	26.26	0.7920	—	—
Omni-SR [52]	2023	32.49	0.8988	28.78	0.7859	27.71	0.7415	**26.64**	0.8018	31.02	0.9151
IFIN [53]	2024	32.27	0.8958	28.68	0.7834	27.62	0.7381	26.17	0.7890	31.06	0.9156
CAMixerSR [54]	2024	32.51	0.8988	28.82	**0.7870**	27.72	0.7416	26.63	0.8012	—	—
GSDAT-Net	**/**	**32.53**	**0.8993**	**28.83**	0.7865	**27.78**	**0.7417**	**26.64**	**0.8020**	**31.08**	**0.9157**

Notes: The bold values indicate the best performance, while the underlined values indicate the second-best performance among all compared methods. The results of SRCNN, CARN, IMDN, LatticeNet, A2N, SwinIR, ESRT, HNCT, PILN, DRSAN, Omni-SR, IFIN, and CAMixerSR are obtained from their original papers. These results are reported under their corresponding official experimental settings. The GSDAT-Net results are evaluated using our implementation with the same benchmark datasets and evaluation metrics.

**Table 4:** Ablation study on the proposed ESA and GAB.

Variant	ESA	GAB	Params (K)	PSNR/SSIM	ΔPSNR
Baseline	×	×	1075	25.71/0.7843	—
Baseline + GAB	×	√	1149	26.52/0.7940	+0.81
Baseline + ESA	√	×	1169	26.59/0.7946	+0.88
Full GSDAT-Net	√	√	1243	26.64/0.8020	+0.93

**Table 5:** Ablation study on Transformer layer selection and ESA placement.

Variant	Transformer Layer	ESA Position	Params (K)	PSNR/SSIM
w/STL	SwinV1	Front	1264	26.56/0.8007
w/S2TL	SwinV2	Back	1243	26.60/0.8011
w/S2TL	SwinV2	Front	1243	26.64/0.8020

**Table 6:** Sensitivity analysis of the Number of RHTBs on Urban100 under ×4 SR.

Number of RHTBs	Params (K)	FLOPs (G)	PSNR/SSIM
4	930	37.5	26.53/0.8000
6	1243	47.92	26.64/0.8020
8	1557	56.2	26.67/0.8026

**Table 7:** Sensitivity analysis of window size on Urban100 under ×4 SR.

Window Size	Params (K)	FLOPs (G)	PSNR/SSIM
4	1238	43.5	26.57/0.8010
8	1243	47.92	26.64/0.8020
16	1254	56.5	26.65/0.8022

**Table 8:** Comparison of parametric quantities and network frames of different reconstruction algorithms with an amplification factor of 4.

Scale	Method	Params (K)	FLOPs (G)	Runtime (ms)	GPU Memory (MB)	Architecture
4	CARN [47]	1592	51.70	4.245	245.2	CNN
	IMDN [48]	715	23.27	4.513	199.7	CNN
	LatticeNet [49]	777	24.82	7.877	195.0	CNN
	SwinIR [27]	897	30.16	26.289	150.7	Transformer
	ESRT [51]	751	33.35	38.883	1641.7	CNN + Transformer
	Omni-SR [52]	792	25.20	19.841	192.8	Transformer
	CAMixerSR [54]	765	18.84	48.792	167.7	Transformer
	GSDAT-Net	1243	47.92	44.446	526.0	CNN + Transformer

## Data Availability

The datasets used in this study, including DIV2K, Set5, Set14, BSD100, Urban100, and Manga109, are publicly available from the sources cited in Section 4.1. The source code, training and evaluation scripts, and random-seed settings are publicly available at https://github.com/chimengkk/GSDAT (accessed on 15 July 2026). No new private dataset was created in this study.
